# Pediatric chronic kidney disease: blood cell count indexes as inflammation markers

**DOI:** 10.1590/2175-8239-JBN-2022-0190en

**Published:** 2023-11-10

**Authors:** Aislander Junio da Silva, Ana Cristina dos Santos Lopes, Ana Paula Lucas Mota, Ana Cristina Simões e Silva, Luci Maria Sant’Ana Dusse, Patrícia Nessralla Alpoim

**Affiliations:** 1 Universidade Federal de Minas Gerais, Faculdade de Farmácia, Departamento de Análises Clínicas e Toxicológicas, Belo Horizonte, MG, Brazil.; 2 Universidade Federal de Minas Gerais, Faculdade de Medicina, Departamento de Pediatria, Belo Horizonte, MG, Brazil.

**Keywords:** Renal Insufficiency, Chronic, Pediatric, Nephrology Inflammation, Blood Cell Counts, White, Biomarkers, Insuficiência Renal Crônica, Pediátrico, Inflamação Nefrológica, Contagem de células sanguíneas brancas, Biomarcadores

## Abstract

**Introduction:**

Chronic kidney disease (CKD) is defined as a progressive decline of kidney functions. In childhood, the main triggering factors are congenital anomalies of the kidneys and urinary tract (CAKUT) and glomerulopathies. Inflammatory responses present challenges for diagnosis and staging, which justifies studies on biomarkers/indexes.

**Aim:**

To define blood cell count indexes and verify their association with pediatric CKD etiology and staging. The included indexes were: Neutrophil-Lymphocyte Ratio (NLR), Derived Neutrophil-Lymphocyte Ratio (dNLR), Lymphocyte-Monocyte Ratio (LMR), Systemic Inflammation Response Index (SIRI), Aggregate Index of Systemic Inflammation (AISI), and Systemic Immune-Inflammation Index (SII).

**Methods:**

We determined the indexes in 52 pediatric CKD patients and 33 healthy controls by mathematical calculation. CKD patients were separated in five groups based on the etiology and staging: Group IA: glomerulopathies at stage 1 or 2; IB: glomerulopathies at stage 3 or 4; IIA: CAKUT at stage 1 or 2; IIB: CAKUT at stage 3 or 4; and III: stages 3 or 4 of other etiologies. In addition, we combined all patients with CKD in one group (IV). Group V was a healthy control group.

**Results:**

Lower values of LMR were observed for groups IB and IIB compared to group V (p = 0.047, p = 0.031, respectively). Increased values of SIRI were found for group III versus group V (p = 0.030). There was no difference for other indexes when the groups were compared two by two.

**Conclusion:**

The LMR and SIRI indexes showed promising results in the evaluation of inflammation, as they correlated with CKD etiologies and specially staging in these patients.

## Resumo

**Introdução:**

Doença renal crônica (DRC) é definida como um declínio progressivo das funções renais. Na infância, os principais fatores desencadeantes são anomalias congênitas dos rins e trato urinário (CAKUT) e glomerulopatias. Respostas inflamatórias apresentam desafios para diagnóstico e estadiamento, o que justifica estudos sobre biomarcadores/índices.

**Objetivo:**

Definir índices de contagem de células sanguíneas e verificar sua associação com etiologia e estadiamento da DRC pediátrica. Os índices incluídos foram: Razão Neutrófilo-Linfócito (NLR), Razão Neutrófilo-Linfócito Derivada (dNLR), Razão Linfócito-Monócito (LMR), Índice de Resposta à Inflamação Sistêmica (SIRI), Índice Agregado de Inflamação Sistêmica (AISI) e Índice de Inflamação Imune Sistêmica (SII).

**Métodos:**

Determinamos índices em 52 pacientes pediátricos com DRC e 33 controles saudáveis por cálculo matemático. Pacientes com DRC foram separados em cinco grupos conforme etiologia e estadiamento: Grupo IA: glomerulopatias em estágio 1 ou 2; IB: glomerulopatias em estágio 3 ou 4; IIA: CAKUT em estágio 1 ou 2; IIB: CAKUT em estágio 3 ou 4; e III: estágios 3 ou 4 de outras etiologias. Além disso, combinamos todos os pacientes com DRC em um grupo (IV). Grupo V foi um grupo controle saudável.

**Resultados:**

Observamos valores menores de LMR nos grupos IB e IIB comparados ao grupo V (p=0,047; p=0,031, respectivamente). Encontramos valores maiores de SIRI para o grupo III versus grupo V (p=0,030). Não houve diferença para outros índices quando os grupos foram comparados dois a dois.

**Conclusão:**

Os índices LMR e SIRI apresentaram resultados promissores na avaliação da inflamação, pois correlacionaram-se com as etiologias da DRC e, principalmente, com o estadiamento desses pacientes.

## Introduction

Chronic kidney disease (CKD) is characterized by the loss of kidney function associated with histological abnormalities for more than three months. In the Brazilian population, CKD has a prevalence of 1.5%, which means that 3 to 6 million Brazilians are carriers of CKD^1–3^. Children are less affected than adults. The incidence of CKD in children varies due to the difficulty in diagnosis and the criteria used to establish disease stage. Undoubtedly, establishing epidemiology data of CKD in pediatric patients is a challenge. The estimated prevalence of CKD in Brazilian children and adolescents is approximately 20 cases per million^3,4,39^.

In adults, CKD etiology frequently involves diabetes mellitus and high blood pressure, whereas in children and adolescents the main triggering factors are congenital anomalies of the kidneys and urinary tract (CAKUT) and glomerular diseases^5–7,40^. A number of factors can cause CAKUT, with genetic factors, renal hypoplasia and dysplasia, and obstructive uropathies being the most common^4–6,38^.

Although there is no definitive cause for the progression of CKD and its complications, the disease seems to be multifactorial. Studies have suggested that hyper-reactivation of inflammatory cells and immunologic responses of neutrophils and lymphocytes may take place through the release of inflammatory cytokines and autoantibodies, which leads to tissue destruction and renal failure^8–10^.

As the inflammatory process is commonly associated with the progression of chronic diseases, markers for early disease diagnosis have been intensively sought after. Changes in the values of hematological parameters, including red cell distribution width (RDW), mean platelet volume (MPV), and plateletcrit (PCT) are known as systemic inflammatory response (SIR) markers^11–13^. These markers have been separately investigated in patients with CKD,^11,14^ and while some seemed to be helpful in predicting the presence or severity of the disease and its association to inflammatory process^13,15,16^, others did not^9,14^.

In recent years, ratios obtained by mathematical calculation using hemogram parameters have been proposed as potential inflammatory markers. These blood cells count indexes include: Neutrophil-Lymphocyte Ratio (NLR) = Neutrophils / Lymphocytes; Derived Neutrophil-Lymphocyte Ratio (dNLR) = Neutrophils / (Global Leukocytes – Neutrophils); Lymphocyte-Monocyte Ratio (LMR) = Lymphocytes/Monocytes; Systemic Inflammation Response Index (SIRI) = Neutrophils × Monocytes / Lymphocytes; Aggregate Index of Systemic Inflammation (AISI) = Neutrophils × Monocytes × Platelets / Lymphocytes and Systemic Immune-Inflammation Index (SII) = Platelets × Neutrophils / Lymphocytes.

The NLR seems to be of prognostic and predictive value, especially in systemic inflammation^11,14^. The increase in NLR values is a risk factor for mortality in inflammatory and infectious diseases, acute coronary syndrome, cardiovascular diseases (CVD), CKD, neoplasms, appendicitis, and COVID-19^11^.

NLR is an excellent index that is correlated to the progression of CKD, presenting an inversely proportional value to the glomerular filtration rate (GFR), where the higher the value of NLR, the lower the GFR^14^. dNLR is a modified NLR, also used to identify systemic inflammation, where higher values are associated with systemic chronic inflammatory diseases and various types of neoplasms such as gastrointestinal, lungs, breast, and kidneys^15^.

LMR is considered a good inflammatory marker that has a low cost and is easy to apply compared to markers such as IL-6, IL-1-b, TNF-α, and thrombomodulin^16^. LMR was proposed not only as a marker of inflammatory processes, but also as a marker of endothelial dysfunction, and has prognostic and predictive value for conditions such as metabolic syndromes, CVD, thyroid dysfunction, liver and kidney diseases, and general chronic inflammation. This marker also shows great applicability in clinical studies involving patients with kidney alterations^12,16^.

Lower LMR values are associated with neoplasms and inflammation, mainly with a worse prognosis^12^. SIRI has prognostic and predictive value in several neoplastic and systemic inflammatory conditions. Higher values indicate greater progression of the inflammatory status in chronic diseases, revealing a worse prognosis^13,17^. Recently, in a multicenter study, AISI was a predictor for severity and intensive care unit admission in COVID-19 patients and SII was a predictor of survival^34^.

In this study, we evaluated NLR, dNLR, LMR, SIRI, AISI, and SII in pediatric CKD patients, aiming to define their use in predicting disease severity. To the best of our knowledge, this is the first time that these markers have been combined in a study.

## Methods

### Ethical Aspects

This study was previously approved by the Ethics and Research Committee of the Federal University of Minas Gerais (CAAE – 07513513.9.0000.5149). The objectives of our study were clearly explained by the researchers to all the participating children and their parents. Clinical data were collected from medical records and biological samples were obtained from each participant.

### Study Population

Pediatric patients were selected at the Pediatric Nephrology Unit of Hospital das Clínicas - UFMG in 2013 and 2014. Initially, medical records from 84 patients were analyzed, and based on the inclusion and exclusion criteria, a total of 52 pediatric patients with pre-dialysis CKD were included in the study.

The inclusion criteria were having a diagnosis of CKD regardless of the etiology in stages between 1 and 4 and being up to 18 years of age. The exclusion criteria were the presence of acute bacterial or virus infection, allergies, fever, or other signs and symptoms suggestive of acute infection, and acute metabolic or clinical alterations at the time of blood cells count and hemogram-derived indexes evaluation. We also excluded patients with glomerulopathies during disease relapses and under the use of corticosteroids or other immunosuppressive medications at the time of blood cells count and hemogram-derived indexes evaluation.

In clinical practice, the diagnosis and staging of CKD is confirmed if the patient presents a GFR below 60 mL/min/1.73m^2^ for three consecutive months combined with alteration of any kidney injury marker or imaging test evidence. The classification of CKD (Table s1) according to stages allows patients to receive more effective treatments^4,18^. Our pediatric patients with CKD are followed-up in a multidisciplinary outpatient service according to a specific protocol. This protocol includes routine exams to evaluate kidney function parameters, hydroelectrolyte and acid base alterations, bone and mineral metabolism, blood counts, iron metabolism, and exclude common viral (cytomegalovirus, Epstein Barr virus, and others based on clinical signs and symptoms) and bacterial infections (urine culture was performed periodically in patients with CAKUT and in any case of fever or other signs and symptoms of urinary tract infection).

Considering the previously established inclusion and exclusion criteria, 52 pediatric patients with CKD were included in the study, and 31 were excluded because they did not fit the profile (Table s2). The medical records were analyzed, and clinical and laboratory data were extracted to create a database.

The control group consisted of 33 age- and sex-matched healthy children and adolescents selected at the Pedagogical Center and at the UFMG Technical College, according to inclusion and exclusion criteria (Table s3).

Finally, our database was composed of 85 subjects, being 52 pediatric patients with CKD and 33 healthy controls. The etiologies of CKD in the patients of this study included CAKUT, glomerulopathies, cystic diseases, and tubulopathies. CKD stages range from 1 to 4. Stages 1 and 2 were considered as early-stage disease and 3 and 4 as advanced stage disease. According to CKD etiology and stage, the patients were distributed in groups (IA, IB, IIA, IIB, III, and V), defined as:

- Group IA (N = 12) - Patients with stage 1 or 2 CKD caused by glomerulopathies;
- Group IB (N = 08) - Patients with stage 3 or 4 CKD caused by glomerulopathies;
- Group IIA (N = 19) - Patients with stage 1 or 2 CKD caused by CAKUT;
- Group IIB (N = 16) - Patients with stage 3 or 4 CKD caused by CAKUT;
- Group III (N = 07) - Patients with CKD in stages 3 or 4 of etiologies other than glomerular disease or CAKUT, such as tubulopathies, cystic diseases, or others.
- Group V (N = 33) - Healthy children.

In addition, a larger group (Group IV - N = 52) composed of all subgroups regardless of CDK etiology was created.

### Blood Cell Count and Hemogram-Derived Indexes

The blood cell count data for pediatric CDK patients were obtained from medical records. For the control group, cell count was performed using the Counter-Coulter T-890 equipment.

The mathematical calculation using hemogram parameters to determine the blood cell count indexes were performed using a Microsoft Excel^®^ spreadsheet.

### Statistical Analysis

Statistical data analysis was performed using SPSS^®^ (version 19.0) and GraphPad Prism^®^ (version 8.02) software. Data normality was tested by the Shapiro-Wilk test. Parametric data were presented as mean and standard deviation. For non-parametric data, median and interquartile ranges were presented. The comparison of continuous variable medians between groups was performed using the Kruskal-Wallis test. Multiple comparisons were performed using Dunn’s post-test. For normal data, Turkey’s test was applied after ANOVA. Values of p ≤ 0.05 were considered significant.

## Results

As causes of CKD in children and adolescents, glomerulopathies in all stages (I, II, III and IV) accounted for 38.45%, with 23.07% of patients in early stages (I or II) and 15.38% in advanced stages (III or IV). The CAKUT group represented 48.06%, with 17.30% of patients in early stages (I or II) and 30.76% in advanced stages (III or IV). The other causes of CKD (tubulopathies, cystic diseases, among others) represented 13.46% of the cases, and all were in more advanced stages (III and IV). Clinical and laboratory data from CKD patients are shown in Tables 1 and 2, respectively.

**Table 1:** Clinical Characteristics of Children and Adolescents Participating in the Study

Parameters	Group IA (N=12)	Group IB (N=8)	Group IIA (N=9)	Group IIB (N=16)	Group III (N=7)	Group IV (N=52)	Group V (N=33)
**Age (years)^b^**	11.50 (4.0)	13.50 (5.0)	14.0 (5.0)	13.50 (5.0)	15.0 (8,0)	14.0 (5.0)	12.0 (5.0)
**Gender^c^** **Male [n(%)]** **Female [N(%)]**	8 (67) 4 (33)	4 (50) 4 (50)	4 (44) 5 (56)	14 (88) 2 (12)	2 (29) 5 (71)	32 (62) 20 (48)	21 (54) 12 (36)
**Height^a^**	1.43 (0.12)	1.45 (0.26)	1.49 (0.15)	1.42 (0.26)	1.42 (0.29)	1.43 (0.21)	1.56 (0.16)
**Weight^a^**	44.28 (16.12)	39.54 (15.23)	43.26 (15.08)	35.75 (17.49)	32.94 (14.31)	39.22 (15.99)	49.58 (16.45)
**BMI (Kg/m^2^)^b^**	20.60 (7.50)	16.65 (8.78)	17.90 (9.10)	15.85 (3.47)	14.20 (3.60)	17.35 (5.65)	19.38 (3.49)
**Blood pressure** **systolic^b^** **diastolic^b^**	100 (18.0) 60,0 (12.0)	105 (19.0) 70.0 (17.0)	110 (17.0) 70.0 (17.0)	110 (25.0) 70.0 (20.0)	110 (26.0) 65.0 (22.0)	109 (16.25) 70.0 (10.0)	-
**Hypotensive medication [N(%)]^c^**	3 (25)	0 (0)	0 (0)	0 (0)	2 (29)	5 (10)	0 (0)
**ACEI [N(%) ]^c^**	8 (67)	4 (50)	4 (44)	10 (63)	4 (57)	30 (58)	0 (0)
**Anemia treatment^c^**	0 (0)	2 (25)	0 (0)	0 (0)	3 (43)	5 (10)	0 (0)

a Normal Distribution: Variables presented as mean and standard deviation.

b Non-normal distribution: Variables presented as median and interquartile range.

c Absolute and relative value.

- Data not available.

Parametric data are presented as mean ± standard deviation. Non-parametric data are presented as median and interquartile range (assessment by the Shapiro-Wilk normality test). BMI: body mass index; CKD: chronic kidney disease; ACE inhibitors: angiotensin-converting enzyme inhibitors; ARA: Angiotensin receptor antagonists.

**Group I:** Glomerulopathies – All stages CKD (n=20); **Subgroup IA**: Glomerulopathies –Stages 1 e 2 CKD (N=12); **Subgroup IB**: Glomerulopathies – Stages 3 e 4 CKD (N=8); **Group II:** CAKUT – All stages CKD (N=25); **Subgroup IIA:** *CAKUT* –Stages 1 e 2 CKD (N=9); **Subgroup IIB:** *CAKUT* – Stages 3 e 4 CKD (N=16); **Group III:** Other – Stages 3 e 4 CKD (N=7); **Group IV:** Stage 1 to 4 CKD (IA, IIA, IB, IIB, III) (N=52); **Group V:** Controls (N=33).

**Table 2:** Laboratory Parameters of Children and Adolescents in Groups IA, IB, IIA, IIB, III and V

Parameters	Subgroup IA (N=12)	Subgroup IB (N=8)	Subgroup IIA (N=9)	Subgroup IIB (N=16)	Group III (N=7)	Group V (N=33)	p
**Red blood cells (nº x 10^6^/mm^3^)^a^**	4.59 (0.39)	4.41 (1.27)	4.39 (0.42)	4.39 (0.68)	4.02 (0.66)	4.75 (0.44)	0.095
**Hemoglobin (g/dL)^a^**	13.9 (1.24)	12.08 (2.32)	12.57 (0.99)	12.24 (1.22)	11.78 (1.76)	13.46 (1.17)	0.006
**Hematocrit (%)^a^**	39.01 (3.68)	35.83 (6.71)	37.77 (2.38)	37.34 (4.18)	35.90 (6.05)	39.99 (3.92)	0.069
**Platelets (nº x 10^3^/mm^3^)^b^**	314,50 (103,25)	269,00 (427,75)	241,00 (76,00)	244,00 (56,50)	258,00 (198,50)	282,00 (72,25)	0,146
**Global de leukocytes (nº x 10^3^/mm^3^)^b^**	5,97 (3,90)	13,40 (7,20)	6,04 (2,85)	2,80 (2,52)	12,54 (11,48)	5,70 (1,90)	0,257
**Neutrophil (nº x 10^3^/mm^3^)^b^**	2.40 (1.79)	11.10 (7.69)	3.59 (2.29)	2.70 (1.70)	9.11 (11.02)	2.81 (1.58)	0.094
**Lymphocyte (nº x 10^3^/mm^3^)^b^**	2.65 (1.29)	2.69 (2.51)	2.00 (1.21)	2.37 (2.10)	2.54 (1.14)	2.41 (0.78)	0.566
**Monocyte (nº x 10^3^/mm^3^)^b^**	0.60 (0.60)	0.60 (0.10)	0.3 (0.2)	0.35 (0.22)	0.45 (0.10)	0.20 (0.10)	0.002
**Eosinophil (nº x 10^3^/mm^3^)^b^**	0.24 (0.19)	0.46 (0.52)	0.32 (0.34)	0.29 (0.40)	0.33 (0.29)	0.14 (0.20)	0.235
**GFR (mL/min/1.73m^2^)^a^**	-	26.37 (15.79)	85.33 (23.91)	33.57 (14.64)	36.50 (11.50)	-	<0.001
**Urea (mg/dL)^a^**	-	83.25 (26.04)	40.50 (10.19)	102.8 (43.85)	90.0 (10.68)	-	<0.001
**Creatinine (mg/dL)^a^**	-	3.67 (1.71)	1.15 (0.37)	3.02 (1.18)	2.59 (0.95)	-	<0.001
**Uric acid (mg/dL)^a^**	-	5.82 (1.49)	6.87 (1.71)	7.11 (1.32)	6.17 (1.25)	-	<0.001
**Sodium (mmol/L)^a^**	-	141.00 (3.16)	140.83 (2.64)	142.10 (2.60)	139.00 (1.83)	-	0.282
**Potassium (mmol/L)^a^**	-	4.41 (1.04)	4.72 (0.34)	5.19 (0.60)	4.93 (0.59)	-	0.041
**Chlorine (mmol/L)^a^**	102.57 (1.90)	106.00 (5.65)	-	106.00 (1.41)	-	-	0.008
**Phosphorus (mg/dL)^b^**	5.10 (1.00)	5.25 (0.0)	-	5.40 (0.29)	-	-	0.644
**Calcium (mg/dL)^a^**	9.55 (0.43)	10.00 (0.56)	-	9.45 (0.21)	-	-	0.081
**Magnesium (mg/dL)^b^**	1.90 (0.50)	1.75 (0.0)	-	-	-	-	0.477
**PTH (pg/mL)^b^**	-	252.00 (418.13)	62.55 (43.53)	176.00 (188.30)	132.50 (17.00)	-	0.006
**Total proteins (g/dL)^a^**	6.56 (1.22)	-	-	7.73 (0.38)	-	-	0.154
**Albumin (g/dL)^a^**	4.17 (0.38)	4.15 (0.07)	-	3.85 (0.21)	-	-	0.484
**Total cholesterol (mg/dL)^b^**	-	161.00 (102.00)	155.50 (35.00)	150.00 (75.00)	166.00 (38.00)	-	0.386
**LDL (mg/dL)^a^**	-	82.85 (12.82)	89.98 (13.07)	78.65 (9.98)	76.50 (38.89)	-	0.442
**HDL (mg/dL)^a^**	-	52.00 (15.66)	45.50 (11.02)	55.14 (26.55)	56.50 (0.71)	-	0.526
**Triglycerides (mg/dL)^b^**	-	95.00 (519.25)	80.50 (87.50)	89.00 (105.00)	165.00 (80.00)	-	0.228

a Normal Distribution: Variables presented as mean and standard deviation.

b Non-normal distribution: Variables presented as median and interquartile range.

c Absolute and relative value.

- Data not available.

Parametric data are presented as mean ± standard deviation (assessment by ANOVA test). Non-parametric data are presented as median and interquartile range (assessment by the Kruskall wallis test).

**Group I:** Glomerulopathies – All stages CKD (n=20); **Subgroup IA**: Glomerulopathies –Stages 1 e 2 CKD (N=12); **Subgroup IB**: Glomerulopathies – Stages 3 e 4 CKD (N=8); **Group II:** CAKUT – All stages CKD (N=25); **Subgroup IIA:** *CAKUT* –Stages 1 e 2 CKD (N=9); **Subgroup IIB:** *CAKUT* – Stages 3 e 4 CKD (N=16); **Group III:** Other – Stages 3 e 4 CKD (N=7); **Group IV:** Stage 1 to 4 CKD (IA, IIA, IB, IIB, III) (N=52); **Group V:** Controls (N=33).

Table 3 shows the indexes medians in the different groups of participants (IA, IB, IIA, IIB, III and V) and Table 4 shows the indexes in patients with CKD in stages 1 to 4 (group IV) and healthy controls (V).

**Table 3:** Blood Count Derived Indexes of the Evaluated Groups

Groups Indexes	Group IA (N=12)	Group IB (N=8)	Group IIA (N=9)	Group IIB (N=16)	Group III (N=7)	Group V (N=33)	P
**NLR**	1.0 (0.79)	2.43 (10.17)	1.30 (1.27)	1.28 (0.81)	2.84 (2.27)	1.13 (0.64)	0.022*
**dNLR**	0.70 (0.60)	1.90 (5.28)	0.90 (0.85)	0.90 (0.53)	1.85 (0.25)	0.90 (0.45)	0.099*
**LMR**	3.70 (3.60)	4.60 (5.10)^a^	6.10 (11.25)	6.70 (2.95)^b^	5.30 (3.97)	11.00 (4.50)^a b^	P<0.001*
**SIRI**	0.90 (0.80)	1.65 (6.25)	0.30 (0.60)	0.50 (0.52)	0.25 (1.0)^c^	0.20 (0.10)^c^	0.003*
**AISI**	241.40 ( - )	484.50 (1207.43)	67.70 (142.75)	120.10 (128.05)	236.55 (515.47)	73.30 (53.55)	0.031*
**SII**	308.30 ( - )	721.45 (1943.50)	475.60 (349.85)	344.70 (238.05)	552.50 (1461.20)	310.40 (215.15)	0.258*

* p < 0.05 (comparison by Kruskal Wallis).

a Dunn post-test p=0.047

b Dunn post-test p=0.031

c Dunn post-test p=0.030

Non-parametric data are presented as median (interquartile range).

Group IA: Glomerulopathies – CKD stages 1 and 2 (N=12); Group IB: Glomerulopathies – CKD stages 3 and 4 (N=8); Group IIA: CAKUT – CKD stages 1 and 2 (N=9); Group IIB: CAKUT – CKD stages 3 and 4 (N=16); Group III: Other etiologies – CKD stages 3 and 4 (N=7); Group V: Control (N=33). NLR: Neutrophil-Lymphocyte Ratio; dNLR: Neutrophil-lymphocyte-derived ratio; LMR: Lymphocyte-Monocyte Ratio; SIRI: Systemic Inflammation Response Index; AISI: Aggregate Index of Systemic Inflammation or Aggregate Index of Systemic Inflammation; SII: Systemic Immune-Inflammation Index or Index of Systemic Immune-Inflammation.

**Table 4:** Blood Count Derived Indexes of Pediatric Patients with CKD and Control Group

Indexes Groups	Group IV (N=52)	Group V (N=33)	P
**NLR**	1.43 (1.10)	1.13 (0.64)	0.104*
**dNLR**	1.05 (0.83)	0.90 (0.45)	0.463*
**LMR**	6.00 (3.88)	11.00 (4.50)	P< 0.001*
**SIRI**	0.60 (0.73)	0.20 (0.10)	0.001*
**AISI**	167.50 (194.70)	73.30 (53.55)	0,007*
**SII**	353.60 (301.35)	310.40 (215.15)	0.442*

* p < 0.05 (Mann-Whitney comparison).

Non-parametric data are presented as median (interquartile range)

Group IV: CKD group stage 1 to 4 (N=52); Group V: Control (N=33). NLR: Neutrophil/lymphocyte ratio; dNLR: Derived Neutrophil-Lymphocyte Ratio; LMR: Lymphocyte-Monocyte Ratio; SIRI: Systemic Inflammation Response Index; AISI: Aggregate Index of Systemic Inflammation or Aggregate Index of Systemic Inflammation; SII: Systemic Immune-Inflammation Index or Index of Systemic Immune-Inflammation.

**Figure 1:**
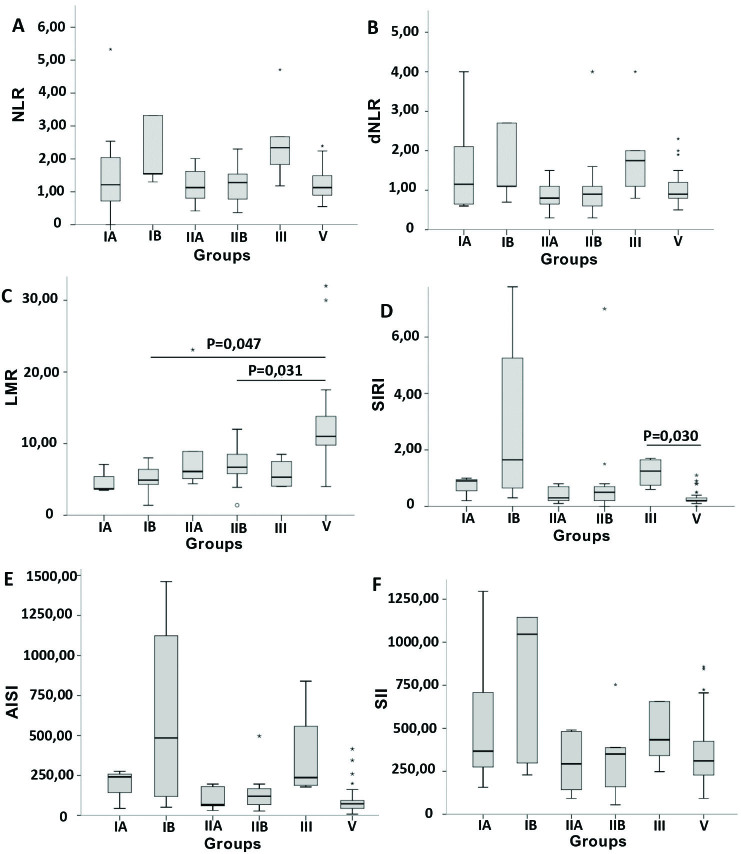
A) NLR values in the groups; B) dNLR values in the groups; C) LMR values in the groups; D) SIRI values in groups; E) AISI values in groups; F) SII values in groups. Group IA: Glomerulopathies – CKD stages 1 and 2 N = 12); Group IB: Glomerulopathies – CKD stages 3 and 4 (N = 8); Group IIA: CAKUT – CKD stages 1 and 2 (N = 9); Group IIB: CAKUT – CKD stages 3 and 4 (N = 16); Group III: Other etiologies – CKD stages 3 and 4 (N = 7); Group V: Control (N = 33). NLR: Neutrophil-Lymphocyte Ratio; dNLR: Neutrophil-lymphocyte-derived ratio; LMR: Lymphocyte-Monocyte Ratio; SIRI: Systemic Inflammation Response Index; AISI: Aggregate Index of Systemic Inflammation or Aggregate Index of Systemic Inflammation; SII: Systemic Immune-Inflammation Index or Index of Systemic Immune-Inflammation.

The distribution of the median values of the indexes (NLR, dNLR, LMR, SIRI AISI and SII) for the six groups (IA, IB, IIA, IIB, III and V) is show in Figure 1.

**Figure 2:**
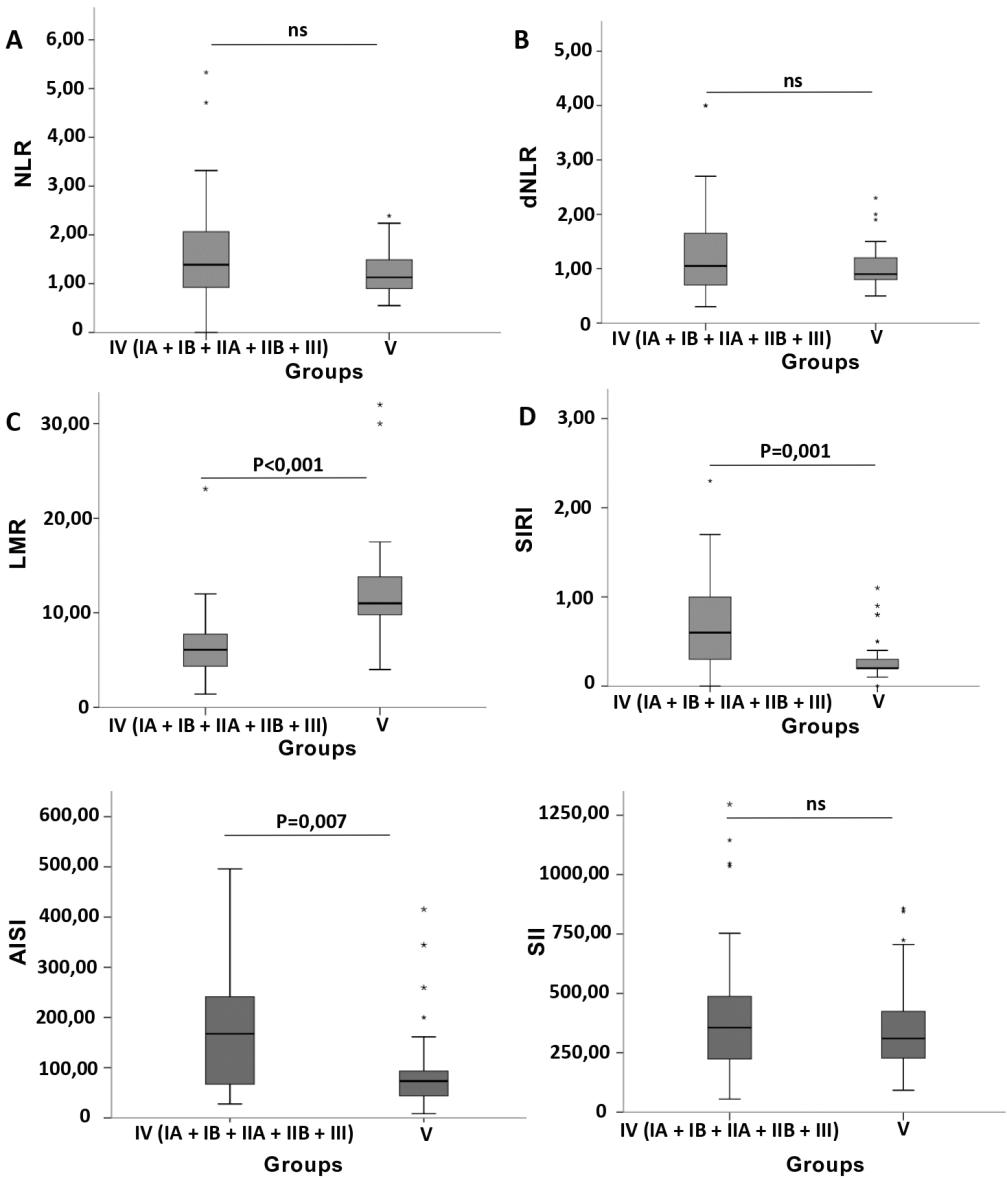
A) NLR values in the groups; B) dNLR values in the groups; C) LMR values in the groups; D) SIRI values in groups; E) AISI values in groups; F) SII values in groups. Group IV: (IA, IB, IIA, IIB, III) CKD Group (N = 52); Group V: Control (N = 33). NLR: Neutrophil-Lymphocyte Ratio; dNLR: Neutrophil-lymphocyte-derived ratio; LMR: Lymphocyte-Monocyte Ratio; SIRI: Systemic Inflammation Response Index; AISI: Aggregate Index of Systemic Inflammation or Aggregate Index of Systemic Inflammation; SII: Systemic Immune-Inflammation Index or Index of Systemic Immune-Inflammation.

A difference was found for NLR among the six groups (P = 0.022). We also performed comparisons between two groups at a time (group 1 vs. group 2, group 1 vs. group 3, and so on). However, no difference was observed between pairs of groups. There was no difference in dNLR among the six groups studied (P = 0.099). For LMR, a difference was observed when comparing the six groups (P < 0.001). In the comparisons between two groups at a time, the LMR was lower when comparing the IB versus V and IIB versus V groups (P = 0.047 and P = 0.031, respectively). For SIRI, the comparison revealed differences among the six groups (P = 0.003), with higher values in group III compared to group V (P = 0.030). For AISI, in the comparison among the six groups, a difference was observed (P = 0.031), but the pair-wise comparison did not show any difference. Finally, no difference was found when comparing the six groups for SII (P = 0.258). The distribution of NLR, dNLR, LMR, SIRI, AISI, and SII values for the group of patients with CKD and healthy controls (IV and V) is shown in Figure 2.

The LMR, SIRI and AISI indexes were significantly different in the CKD group (IV) and controls (V) (P < 0.001, P = 0.001, P = 0.007, respectively). Medians and interquartile ranges for LMR values were 6.00 (3.88) for group IV and 11.00 (4.50) for group V. The SIRI medians were 0.60 (0.73) and 0.20 (0.10) for groups IV and V, respectively. Similarly, the AISI medians were 167.50 (194.70) and 73.70 (53.55) for groups IV and V, respectively. There was no difference for NLR, dNLR, and SII (P = 0.104, P = 0.463, and P = 0.442 respectively).

## Discussion

In the present study, all CKD groups presented red blood cell parameters within the reference value, including groups in advanced stages (for ages between 1 and 6 years, the reference values for red blood cells, hemoglobin, and hematocrit are 4.5 ± 0.6 × 10^12^/L; 12.6 ± 1.5 g/dL; 37 ± 3%; from 6 to 12 years, 4.5 ± 0.6 × 10^12^/L; 12.5 ± 1.5 g/dL; 40 ± 3%; and over 12 years of age, 5.0 ± 1.5 × 10^12^/L; 14.0 ± 2 g/dL; 35 ± 10%, respectively)^19^. It should be noted that 16% of patients at an advanced stage were being treated with recombinant erythropoietin (EPO) to prevent anemia. Likewise, the platelet count for all groups was within the reference value (between 150 and 450 × 10^3^/mm^3^)^19^.

Regarding global leukocyte count, glomerulopathies and early-stage CAKUT groups and the control group (IA, IIA and V) showed normal values: 5.0 to 13.0 × 10^9^/L for children aged 2 to 12 years and 4.0 to 11.0 × 10^9^/L for children 12 or older. The differential leukocyte count revealed that the groups with CKD caused by glomerulopathies in advanced stages and other etiologies (IB and III) had neutrophilia, with values of 11.10 and 9.11 × 10^3^/mm^3^, respectively (reference value is 2 to 7 × 10^3^/mm^3^)^19^. Inflammation in chronic diseases is characterized by the predominance of vascular phenomena with increased permeability and increased number of neutrophils, which play their role in the inflammatory site through diapedesis^20^. Through this process, cells migrate from the bloodstream to the site of inflammation where they are required to act in an inflammatory/regenerative response^9,21^.

Monocytes are highly reactive cells in the inflammatory processes. In tissues, they act on either M1 or M2 immune responses, and depending on the stimulus they can exacerbate or attenuate inflammation^16^. The median values for monocyte count of each group remained within the reference values, from 0.2 to 1.0 × 10^3^/mm^3 22^, as for lymphocytes and eosinophils, from 1.0 to 3.0 × 10^3^/mm^3^ and from 0.02 to 0.5 × 10^3^/mm^3^, respectively^22^.

Usually, the kidney function status is assessed by markers such as GFR, creatinine, urea, and uric acid. Changes in these parameters are suggestive of impaired kidney function^3,18^.

GFR is useful to classify CKD, and differences in GFR were observed among the evaluated groups. All CKD groups had GFR averages lower than the reference values, which allowed stage classification of the studied CKD patients. Groups in advanced stages of CKD (IB, IIB and III) had lower GFR, corroborating the findings in the literature^3,18,23,24^.

All CKD groups had serum urea levels higher than the reference values (8 to 36 mg/dL)^25^, and those with advanced stage CKD presented the highest levels. Serum creatinine levels were also high in all CKD groups, and groups in advanced stages (IB, IIB and III) showed even higher levels.

It is known that uric acid accumulates in the blood in CKD, raising its circulating levels^25,26^. In agreement with the literature, values above the reference values (0.5 to 6mg/dL) were obtained for the CAKUT and other etiologies groups.

The regulation of the hydroelectrolytic balance results from the gain and loss of electrolytes/water ratio. If sodium (Na^+^) intake is high, its reabsorption by the kidney tubules is reduced and, consequently, a higher volume of urine is produced. Chlorine (Cl^–^) also participates in this process, but a smaller amount of chlorine is eliminated. Other ions, such as potassium (K^+^), Ca^2+^, and phosphate (PO_4_^3–^) have a role in this process. However, kidney injuries result in changes in the function of the kidney tubules, compromising the hydroelectrolytic balance^27,28^. All CKD groups presented Na^+^ and K^+^ levels within the reference values (132 to 145 mEq/L and 3.5 to 5.1 mEq/L, respectively)^25^. This is probably because CKD patients from the second stage onwards make periodic determinations of these ions, and intervention measures are promptly adopted when alterations are detected to restore the hydroelectrolytic balance^18,36^. For all evaluated groups, the mean chlorine levels were within the reference range (97 to 106 mEq/L)^25^.

It is known that the balance of calcium levels is controlled by the action of parathyroid hormone (PTH), produced by the parathyroid glands. In cases of calcium loss or reduction, PTH acts at the kidney level, promoting Ca^2+^ reabsorption and stimulating its release from the bone tissue. PTH also acts on the kidneys by reducing the reabsorption of PO_4_^3–27,28^.

All CKD patients had calcium levels within the reference values (8.8 to 10.8 mg/dL)^25^, since they are monitored and treated when necessary^19,36^. However, PTH levels were very high compared to reference values (18.5 to 88.0 pg/mL), especially in groups of advanced CKD stages. This is because kidney diseases cause an alteration in calcium levels due to its loss in the urine, which results in a greater release of PTH, increasing calcium circulation levels as an attempt to avoid the loss of calcium^29^.

It is known that in CKD, especially when caused by glomerulopathies, there is a change in the lipid profile due to kidney losses and consequent stimulation of the hepatic production of lipoproteins^28^. Nonetheless, all studied groups had medians of total cholesterol within reference values (<170 mg/dL)^25^.

### Blood Cell Count Indexes

All advanced stages CKD groups had higher NLR values compared to the control group (V). This demonstrates that progression of CKD with deterioration of kidney function is associated with inflammation^11,14^. However, there was no difference when comparing the groups of patients with CKD (all etiologies in stages 1 to 4) (IV) to the control group (V). For dNLR and SII, there was no difference when comparing the groups with CKD among themselves or in relation to the controls.

LMR had a lower value in CKD patients compared to the control group, which suggests a higher degree of inflammation in the CKD patients. For patients with advanced stage glomerulopathies (IB) and advanced stage CAKUT (IIB), the LMR showed significantly lower values compared to the control group (V), suggesting that the higher inflammatory level, the greater the progression of CKD^30,31,35,37^. The LMR was lower in the groups with early and advanced stage glomerulopathies (IA and IB). It is known that in glomerulopathies, the harmful inflammatory process affects the glomerular endothelial cells and, therefore, LMR may be associated with endothelial dysfunctions^16^.

Regarding SIRI, all groups (early and advanced stages glomerulopathies, early and advanced stages CAKUT and other etiologies CKD) had values higher than the control group (V). However, the difference was observed only between CKD patients with other etiologies at an advanced stage (III) and the control group (V). When comparing the group of patients with CKD (IV) to the control group (V), SIRI was significantly increased in the CKD group IV. It is known that the greater the progression of CKD and deterioration of kidney function, the greater the parenchymal inflammatory process^32,33,37^. This index reveals a relationship between neutrophils, lymphocytes, and monocytes and allows evaluating the inflammatory action. It is known that neutrophils are able to promote inflammation in the microenvironment (renal parenchyma) and inhibits lymphocyte activity with suppression of the regulatory response of T cells and activation of macrophage cells. Thus, with a higher numerator (neutrophils and monocytes) and a lower denominator (lymphocytes), the SIRI score increases, reflecting the inflammatory status^13,17^.

AISI had a trend for higher values in advanced stages of CKD (IB and IIB) compared to early stages in each etiology. As an inflammation marker, AISI is associated with death. Higher AISI values indicate reduced survival probability in patients with COVID-19 and idiopathic pulmonary fibrosis, and in the latter, it is possible to determine the severity and stage of the disease^34^, even if no difference was found when comparing two groups at a time. Therefore, it is possible to observe the difference by comparing CKD and control groups, revealing the inflammatory state. However, AISI determination was not considered as an inflammation marker in patients with CKD, both in children and adults.

## Conclusion

Multiple factors play a role in CKD, such as genetics, lifestyle, age, immunological condition, oxidative stress, uremic status, and infections, which characterizes it as a multifactorial and heterogeneous disease. The inflammatory response in the renal parenchyma is complex and with dualities, depending on intrinsic factors of each patient.

Thus, in the pediatric age, this complexity is added to other peculiarities, like child growth, endocrine profile, environmental and social adaptation, emotional and organic stresses, as well as inflammatory responses. The data obtained in this pioneering study, involving Brazilian children and adolescents with CKD, allow us to infer that blood cell count-derived indexes, such as LMR and SIRI, are promising for determining the inflammatory status of CKD children and adolescents according to etiology and stage.

## Supplementary Material

The following online material is available for this article:

Table s1 - Classification of stages of Chronic Kidney Disease.

Table s2 - Inclusion and exclusion criteria for children with CKD.

Table s3 - Inclusion and exclusion criteria for control group.

## References

[1] Lee SA, Noel S, Sadasivam M, Hamad AR, Rabb H (2017). Role of immune cells in acute kidney injury and repair. Nephron Clin Pract..

[2] Marinho AW, Penha AP, Silva MT, Galvão TF (2017). Prevalence of chronic renal disease among Brazilian adults: a systematic review. Cad Saude Colet..

[3] Kidney Disease: Improving Global Outcomes (KDIGO) CKD Work Group (13). Kidney Inter Suppl..

[4] Kaspar CD, Bholah R, Bunchman TE (2016). A review of pediatric chronic kidney disease. Blood Purif..

[5] Ingelfinger JR, Kalantar-Zadeh K, Schaefer F (2016). World Kidney Day Steering Committee. In time: averting the legacy of kidney disease – focus on childhood. Rev Paul Pediatr..

[6] Belangero VM, Prates LC, Watanabe A, Schvartsman BS, Nussenzveig P, Cruz NA (2018). Prospective cohort analyzing risk factors for chronic kidney disease progression in children. J Pediatr (Rio J)..

[7] Thomé FS, Sesso RC, Lopes AA, Lugon JR, Martins CT (2019). Brazilian chronic dialysis survey 2017. J Bras Nefrol..

[8] Vianna HR, Soares BM, Tavares MS, Teixeira MM, Silva AC (2011). Inflammation in chronic kidney disease: the role of cytokines. Brazilian Journal of Nephrology..

[9] Akchurin OM, Kaskel F (2015). Update on inflammation in chronic Kidney Disease. Blood Purif..

[10] Su H, Lei CT, Zhang C (2017). Interleukin-6 signaling pathway and its role in kidney disease: an update. Front Immunol..

[11] Liu Y, Du X, Chen J, Jin Y, Peng L, Wang HHX (2020). Neutrophil-to-lymphocyte ratio as an independent risk for mortality in hospitalized patients with COVID-19. J Infect..

[12] Goto W, Kashiwagi S, Asano Y, Takada K, Takahashi K, Hatano T (2018). Predictive value of lymphocyte-to-monocyte ratio in the preoperative setting for progression of patients with breast cancer. BMC Cancer..

[13] He Q, Li L, Ren Q (2021). The Prognostic value of preoperative systemic inflammation response index (SIRI) in patients with High-grade glioma and the establishment of a nomogram. Front Oncol..

[14] Rios DR, Pinheiro MB, Oliveira WV, Gomes KB, Carvalho AT, Martins-Filho OA (2017). Cytokine signature in end-stage renal disease patients on hemodialysis. Dis Markers..

[15] Eskiizmir G, Uz U, Onur E, Ozyurt B, Cikrikci GK, Sahin N (2019). The evaluation of pretreatment neutrophil-lymphocyte ratio and derived neutrophil-lymphocyte ratio in patients with laryngeal neoplasms. Rev Bras Otorrinolaringol (Engl Ed)..

[16] Balta S, Demirer Z, Aparci M, Yildirim AO, Ozturk C (2016). The lymphocyte-monocyte ratio in clinical practice. J Clin Pathol..

[17] Zheng Y, Chen Y, Chen J, Chen W, Pan Y, Bao L (2019). Combination of systemic inflammation response index and platelet-to-lymphocyte ratio as a novel prognostic marker of upper tract urothelial carcinoma after radical nephroureterectomy. Front Oncol..

[18] Levey AS, Eckardt KU, Tsukamoto Y, Levin A, Coresh J, Rossert J, De Zeeuw D, Hostetter TH, Lameire N, Eknoyan G (2005). Definition and classification of chronic kidney disease: a position statement from Kidney Disease. Kidney Int..

[19] Bain BJ, Bates I, Laffan MA (2017). Practical haematology.

[20] Gavins FN, Hickey MJ (2012). Annexin A1 and the regulation of innate and adaptive immunity. Front Immunol..

[21] Norlander AE, Saleh MA, Madhur MS (2014). CXCL16: a chemokine causing chronic kidney disease. Hypertension..

[22] Zinelluv A, Collu C, Nasser M, Paliogiannis P, Mellino S, Zinelli E (2021). The Aggregate Index of Systemic Inflammation (AISI): a novel prognostic biomarkers in idiopathic pulmonary fibrosis. J Clin Med..

[23] Kirsztajn GM, Salgado Fo N, Draibe SN, Netto MV, Thomé FS, Souza E (2014). Fast Reading of the KDIGO 2012: guidelines for evaluation and management of chronic kidney disease in clinical practice. J Bras Nefrol..

[24] Krane V, Wanner C (2011). Statins, inflammation and kidney disease. Nat Rev Nephrol..

[25] Gaw A, Murphy MJ, Srivastava R, Cowan RA, O’Reilly DS (2015). Clinical biochemistry..

[26] Bastos MG, Kirsztajn GM (2011). Chronic kidney disease: importance of early diagnosis, immediate referral and structured interdisciplinary approach to improve outcomes in patients not yet on dialysis. Brazilian Journal of Nephrology..

[27] Hall JE, Guyton AC (2020). Medical physiology..

[28] Rennke HG, Denker BM (2009). Renal pathophysiology: the essentials..

[29] Becherucci F, Roperto RM, Materassi M, Romagnani P (2016). Chronic kidney disease in children. Clin Kidney J..

[30] Guzzi F, Cirillo L, Roperto RM, Romagnani P, Lazzeri E (2019). Molecular mechanisms of the acute kidney injury to chronic kidney disease transition: an updated view. Int J Mol Sci..

[31] Kimmel PL, Rosenberg ME (2020). Chronic Renal Disease.

[32] Gungor O, Unal HU, Guclu A, Gezer M, Eylleten T, Guzel FB (2017). IL-33 and ST2 levels in chronic kidney disease: associations with inflammation, vascular abnormalities, cardiovascular events, and survival. PLoS One..

[33] Chen WY, Li LC, Yang JL (2017). Emerging roles of IL-33/ST2 axis in renal diseases. Int J Mol Sci..

[34] Hamad AH, Aly MM, Abdelhameid MA, Ahmed SA, Shaltout AS, Abdel-Moniem AE (2021). Combined blood indexes of systemic inflammation as a mirror to admission to intensive Care Unit in COVID-19 patients: a multicentric study. J Epidemiol Glob Health..

[35] Soares CM, Diniz JS, Lima EM, Oliveira GR, Canhestro MR, Colosimo EA (2009). Predictive factors of progression to chronic kidney disease stage 5 in a predialysis interdisciplinary programme. Nephrol Dial Transplant..

[36] Park KS, Hwang YJ, Cho MK, Ko CW, Ha S, Kang HG (2012). Quality of life in children with end-stage renal disease based on a PedsQL ESRD module. Pediatr Nephrol..

[37] Nogueira PC, Paz IP (2016). Signs and symptoms of developmental abnormalities of the genitourinary tract. J Pediatr (Rio J)..

[38] Chevalier RL, Thornhill BA, Forbes MS, Kiley SC (2010). Mechanisms of renal injury and progression of renal disease in congenital obstructive nephropathy. Pediatr Nephrol..

[39] Nogueira PC, Feltran LS, Camargo MF, Leão ER, Benninghover JR, Gonçalves NZ (2011). Estimated prevalence of childhood end-stage renal disease in the state of São Paulo. Rev Assoc Med Bras (1992)..

[40] Martelli A (2012). The role of the kidney in regulating arterial blood pressure Blood Pressure. Nat Rev Nephrol..

