# Supplementary material for: Pediatric chronic kidney disease: blood cell count indexes as inflammation markers
Source: J Bras Nefrol. 2023 Nov 10;45(4):458–69. doi: 10.1590/2175-8239-JBN-2022-0190en (PMC10726671; doi:10.1590/2175-8239-JBN-2022-0190en)
Supplement: Supplementary file 1 [file 2175-8239-jbn-2022-0190-s1.pdf]

Braz. J. Nephrol. (São Paulo) 2023. Ahead of print
https://doi.org/10.1590/2175-8239-JBN-2022-0190en

Supplementary Material to "Pediatric chronic kidney disease: blood cell count indexes as inflammation markers"

Table s1 - Classification of stages of Chronic Kidney Disease

**CKD stages**	**Description**	**GFR mL/min/1.73m^2**
1	Normal or increased GFR	Above 90
2	Kidney damage with slightly reduced GFR	Between 60 and 89
3 a	Kidney damage with mild to moderately reduced GFR	Between 45 and 59
3 b	Kidney damage with moderately to severely reduced GFR	Between 30 and 44
4	Kidney damage with markedly reduced GFR	Between 15 and 29
5	Kidney failure	Below 15
