# Supplementary material for: Pediatric chronic kidney disease: blood cell count indexes as inflammation markers
Source: J Bras Nefrol. 2023 Nov 10;45(4):458–69. doi: 10.1590/2175-8239-JBN-2022-0190en (PMC10726671; doi:10.1590/2175-8239-JBN-2022-0190en)
Supplement: Supplementary file 2 [file 2175-8239-jbn-2022-0190-s2.pdf]

Braz. J. Nephrol. (São Paulo) 2023. Ahead of print
https://doi.org/10.1590/2175-8239-JBN-2022-0190en

Supplementary Material to "Pediatric chronic kidney disease: blood cell count indexes as inflammation markers"

Table s2 - Inclusion and exclusion criteria for children with CKD

Inclusion criteria for children with CKD	Exclusion criteria for children with CKD
- Diagnosed glomerular disease (Group I)	- Be over 18 years old
- CKD due to glomerular disease in stages 1, 2, 3 or 4 (Group I)	- Be less than 2 years old or underweight
- Be under 18 years old at the time of biological sample collection (Group I)	- CKD with GFR below 15 ml/min/1.73m^2 and/or on hemodialysis
- CKD stage 1 or 2 due to glomerular disease (Subgroup IA)	- Children with infectious diseases: (Cytomegalovirus, Epstein Barr virus, bacterial infection) and others based on clinical signs and symptoms
- CKD stage 3 or 4 due to glomerular disease (Subgroup IB)	- Children with an infectious and/or inflammatory process on the date of collection or up to three weeks previously
- Diagnosed CAKUT (Group II)	- Diabetes mellitus
- Be under 18 years old at the time of biological sample collection (Group II)	- Children with other diseases, such as cancer, autoimmune diseases, liver disease, hormonal disorders and anemia
- CKD stage 1 or 2 due to CAKUT (Subgroup IIA)	- Children with platelet or clotting disorders
- CKD stage 3 or 4 due to CAKUT (Subgroup IIB)	- Using multivitamin drugs or contraceptive hormones
Have a diagnosis of CKD stages 3 or 4 of etiologies other than glomerular disease and CAKUT (Group III)	-Patients under corticosteroids or immunosuppressive medications at the time of blood cells count and hemogram-derived indexes
- Be under 18 years old at the time of biological sample collection (Group III)	-
