# Supplementary material for: Pediatric chronic kidney disease: blood cell count indexes as inflammation markers
Source: J Bras Nefrol. 2023 Nov 10;45(4):458–69. doi: 10.1590/2175-8239-JBN-2022-0190en (PMC10726671; doi:10.1590/2175-8239-JBN-2022-0190en)
Supplement: Supplementary file 3 [file 2175-8239-jbn-2022-0190-s3.pdf]

Braz. J. Nephrol. (São Paulo) 2023. Ahead of print
https://doi.org/10.1590/2175-8239-JBN-2022-0190en

Supplementary Material to "Pediatric chronic kidney disease: blood cell count indexes as inflammation markers"

Table s3 - Inclusion and exclusion criteria for the control group

Inclusion criteria	Exclusion criteria
- Be clinically healthy	- Older than 18 years
- Age and sex matched with CKD participants	- Younger than 2 years or underweight
-	- Presence of any kidney and urinary tract pathology
-	- Presence of infectious diseases, cancer, autoimmune diseases, liver disease, hormonal disorder
-	- Presence of an infectious and/or inflammatory process on the date of collection or up to three weeks previously
-	- Diabetes mellitus or other metabolic diseases
-	- Platelet or coagulation disorders
-	- Using any anti-inflammatory drugs, contraceptive hormones or multivitamins
